# *FUT2,* Secretor Status and *FUT3* Polymorphisms of Children with Acute Diarrhea Infected with Rotavirus and Norovirus in Brazil

**DOI:** 10.3390/v12101084

**Published:** 2020-09-25

**Authors:** Marco André Loureiro Tonini, Débora Maria Pires Gonçalves Barreira, Luciana Bueno de Freitas Santolin, Lays Paula Bondi Volpini, José Paulo Gagliardi Leite, Béatrice Le Moullac-Vaidye, Jacques Le Pendu, Liliana Cruz Spano

**Affiliations:** 1 Laboratory of Virology and Infectious Gastroenteritis, Pathology Department, Health Science Center, Federal University of Espírito Santo, Maruípe, Vitória 1468, ES, Brazil; deborambarreira@yahoo.com.br (D.M.P.G.B.); freitas_lb@yahoo.com.br (L.B.d.F.S.); layspaula90@gmail.com (L.P.B.V.); liliana.spano@ufes.br (L.C.S.); 2 Laboratory of Comparative and Environmental Virology, Oswaldo Cruz Institute, Rio de Janeiro 4365, RJ, Brazil; jpgleite@ioc.fiocruz.br; 3 CRCINA, Inserm, Université de Nantes, F-44000 Nantes, France; beatrice.vaidye@inserm.fr (B.L.M.-V.); jacques.le-pendu@univ-nantes.fr (J.L.P.)

**Keywords:** host susceptibility, histo-blood group antigens (HBGAs), norovirus, rotavirus, secretor status, polymorphisms of *FUT2* and *FUT3*, Lewis

## Abstract

Host susceptibility according to human histo-blood group antigens (HBGAs) is widely known for norovirus infection, but is less described for rotavirus. Due to the variable HBGA polymorphism among populations, we aimed to evaluate the association between HBGA phenotypes (ABH, Lewis and secretor status) and susceptibility to rotavirus and norovirus symptomatic infection, and the polymorphisms of *FUT2* and *FUT3*, of children from southeastern Brazil. Paired fecal-buccal specimens from 272 children with acute diarrhea were used to determine rotavirus/norovirus genotypes and HBGAs phenotypes/genotypes, respectively. Altogether, 100 (36.8%) children were infected with rotavirus and norovirus. The rotavirus P[8] genotype predominates (85.7%). Most of the noroviruses (93.8%) belonged to genogroup II (GII). GII.4 Sydney represented 76% (35/46) amongst five other genotypes. Rotavirus and noroviruses infected predominantly children with secretor status (97% and 98.5%, respectively). However, fewer rotavirus-infected children were Lewis-negative (8.6%) than the norovirus-infected ones (18.5%). *FUT3* single nucleotide polymorphisms (SNP) occurred mostly at the T59G > G508A > T202C > C314T positions. Our results reinforce the current knowledge that secretors are more susceptible to infection by both rotavirus and norovirus than non-secretors. The high rate for Lewis negative (17.1%) and the combination of SNPs, beyond the secretor status, may reflect the highly mixed population in Brazil.

## 1. Introduction

Diarrheal diseases remain a leading cause of morbidity and mortality in children under five years of age, and account for nearly 500,000 deaths annually in the world, especially in low-income countries. Enteric viruses, particularly norovirus and rotavirus, are the major etiological agents of diarrhea worldwide [1,2]. Since 2006, two oral, live-attenuated rotavirus vaccines (Rotarix^®^ and RotaTeq^®^) have been implemented nationally in 98 countries, and their use has substantially reduced global child deaths from diarrheal illness [3,4]. Nevertheless, the effectiveness of current vaccines is lower in low- and middle-income countries compared with higher-income settings [5]. Among other factors, host genetic characteristics have been proposed to explain this difference [6,7].

Rotaviruses are classified into 10 species (A to J), with species A being the major cause of infections in children [8]. Two outer capsid proteins, VP4 (protease-sensitive protein) and VP7 (glycoprotein), are traditionally used to define the rotavirus P and G genotypes, respectively. Currently, 36 G and 51 P genotypes have been identified, among which six G genotypes (G1, G2, G3, G4, G9, and G12) and three P genotypes (P[4], P[6], P[8]) are more prevalent globally [9]. As for noroviruses, they are dually classified based on the complete VP1 (major capsid protein) coding region, corresponding to open reading frame (ORF) 2, and on a partial RNA polymerase (RdRp) coding region located at the 3′-end of the ORF1. Ten norovirus genogroups (GI–GX) are recognized according to the VP1 classification, each being subdivided into genotypes. Genogroups GI and GII that encompass more than 30 genotypes are the main causes of human infection. Among them, the GII.4 genotype is responsible for the majority of norovirus outbreaks and sporadic cases worldwide [10,11]. The *RpRd* gene classifies noroviruses into eight P-groups and 62 P-types [12].

Both viruses recognize and bind to histo-blood group antigens (HBGAs) which act as host attachment factors for viral infection on enteric epithelial cells [13,14,15]. Human HBGAs are highly polymorphic carbohydrates expressed on mucosal epithelial cells and the surface of erythrocytes and in biological fluids such as saliva and milk. Their synthesis occurs by the sequential addition of monosaccharides to precursor disaccharides by four major glycosyltransferases: α-1, 2-fucosyltransferase (FUT-2), α-1, 3 fucosyltransferase (FUT3) and A and B enzymes, which are coded by three main HBGA gene families, resulting in ABO (A, B and/or H antigens), Lewis (Lewis a, b, x and/or y antigens) and secretor/non-secretor HBGA phenotypes [16]. The expression of these phenotypes are genetically determined and depend on individual HBGA genotypes, which are influenced by the composition and genetic diversity of the human population [6]. A mutation on both alleles of the *FUT2* gene leads to inactivation of the corresponding FUT2 enzyme, and individuals possessing such polymorphism are termed non-secretors. Similarly, individuals with null alleles for the *FUT3* gene do not produce Lewis antigens, and are denominated Lewis negative [6].

Genetic susceptibility to norovirus infection mediated by HBGA polymorphisms has been a known phenomenon for decades and, despite solid scientific evidence regarding the association between the norovirus infection, particularly with the predominant GII.4 genotype, and the secretor status, the exact role of the Lewis antigen as a susceptibility mediator remains to be better clarified [17,18,19].

Less than a decade ago, in vitro studies showing the binding of the VP8* domain of the spike protein VP4 of rotavirus to HBGA paved the way for the development of epidemiological evaluations to understand the effect of HBGA differential expression in susceptibility to rotavirus infection [14,15]. These epidemiological studies conducted in different countries including France, Vietnam, Burkina Faso, USA, China, and Spain pointed to the same conclusion that children of the secretor phenotype were significantly more prone to suffer from diarrhea caused by P[8] and P[4] rotavirus compared to the non-secretor [20,21,22,23,24,25]. However, conflicting results were observed in studies conducted with children from Tunisia, where P[8] strains were found infecting both individuals of secretor and non-secretor phenotypes, and more recently in Bangladesh, where the non-secretor children were not protected from diarrhea caused by the P[8] genotype [26,27]. These inconsistencies suggest the need for more epidemiological studies in different populations to better understand the role of HBGA phenotypes in rotavirus infection.

In Brazil, a continental-sized country with a highly genetically admixed population, few studies have analyzed the association between norovirus infection and HBGA phenotypes [28,29] and there are no data yet available regarding rotavirus infection and disease. Therefore, this study aimed to analyze the association between rotavirus and norovirus symptomatic infection and HBGA phenotypes, in addition to *FUT2* and *FUT3* genes polymorphisms, in children from Southeastern Brazil. In the present study, we were able to reinforce the current knowledge that secretors are more susceptible than non-secretors to infection by both rotavirus and norovirus, and that the combination of *FUT3* polymorphisms found may reflect the highly mixed Brazilian population.

## 2. Materials and Methods

### 2.1. Study Population and Samples

Fecal and buccal swabs specimens were collected from 272 children up to 12 years old enrolled during a surveillance study of sporadic cases of acute gastroenteritis carried out in two periods (October 2014 to July 2016 and September 2017 to September 2018) in the Southeastern Brazilian coastal state of Espírito Santo. Among these children, 196 (72.1%) had received rotavirus vaccination, of which 92.9% (182/196) were fully vaccinated (179 received Rotarix^®^ and three RotaTeq^®^) and the other 14 were partially vaccinated with Rotarix^®^. Twenty-seven (10%) children were unvaccinated and for 49 children (18%) the vaccination status was not available. These children were admitted to a pediatric hospital and an urgent care center situated in the city of Vitória (state capital). During their admission, they were invited to participate in the study and samples were obtained after the signature of a consent form by the person responsible for the patient. The study was approved by the Institutional Research Ethics Committee of the Center of Health Sciences of the Federal University of Espírito Santo (N° 1.312.166; 6 November 2015).

The passage of three or more loose or watery stools during a 24 h period with up to 14 days of duration was considered acute diarrhea [30].

### 2.2. Rotavirus and Noroviruses Detection

Viral RNA was extracted from 10% fecal suspensions (*w*/*v*) using the QIAamp^®^ Viral RNA Mini Kit (QIAGEN, Valencia, CA, USA), according to the manufacturer’s instructions. Noroviruses were detected by RT-qPCR using primers and a probe targeting the ORF1/2 junction [31,32]. Rotavirus detection was performed either by an Enzyme immunoassay (ELISA) (Ridascreen^®^, R-Biopharm, Darmstadt, Germany), according to the manufacturer’s recommendation, or by RT-qPCR, with primers and a probe specific to amplifying a conserved region of the non-structural protein 3 gene (*NSP3*) of the virus [33].

### 2.3. Rotavirus P Typing and Noroviruses Genotyping

Rotavirus P types were determined from positive samples with specific primers for a fragment of the *VP4* gene, followed by sequencing [34]. For noroviruses genotyping, positive samples were initially amplified by PCR using a set of primers targeting a 570 bp fragment encompassing the 3′-end ORF1 and 5′-end ORF2 and then sequenced [35,36]. Sequencing for both viruses was carried out with ABI Prism Big Dye Terminator v3.1 Cycle Sequencing Ready Reaction Kit on an ABI Prism 310 Genetic Analyzer (Applied Biosystems, Foster City, CA, USA). The noroviruses’ genotype was ascribed using the Norovirus Automated Genotyping Tool version 2.0, (https://www.rivm.nl/mpf/typingtool/norovirus/) [37]. The 33 VP4 sequences of rotavirus were deposited on GenBank under the accession numbers MT597424 and MT602430-MT602461. As to norovirus, the 46 ORF-1/2 sequences were deposited on GenBank under the accession numbers KY451971-KY451984, MF158177-MF158179, MF158185-MF158199 [38], MT613346-MT613358, and MT626025.

### 2.4. Histo-Blood Group Antigens Phenotyping

HBGAs phenotypes were determined from buccal swabs specimens by enzyme-linked immunosorbent assay (ELISA), as described elsewhere [39]. Buccal swabs immersed in phosphate-buffered saline (PBS) were first boiled for 10 min, and then used at a dilution of 1:50 in a 0.1 M carbonate/bicarbonate buffer (pH 9.6) to coat 96-well microtiter plates (Maxisorp Nunc-Immuno plates, Thermo Scientific, Roskilde, Denmark), which were then incubated overnight at 4 °C. The plates were washed three times with PBS containing 0.05% Tween-20, and blocked with PBS containing 5% nonfat milk (5% milk/PBS) for 1 h at 37 °C. Primary anti-carbohydrate monoclonal antibodies anti-A (3-3A), anti-B (B49), anti-Le^a^ (7LE) and anti-Le^b^ (2-25LE) (Thermo Scientific) diluted at 1:400 in 5% milk/PBS were added and incubated for 1 h at 37 °C. At this step, the lectin UEA-1 (“Ulex Europaeus Agglutinin I”–Vector Laboratories, Burlingame, CA, USA) conjugated with biotin was also used to detect the H antigen. After three washes, peroxidase-conjugated anti-mouse immunoglobulin diluted at 1:2000 in 5% milk/PBS and peroxidase-conjugated Streptavidin (Vector Laboratories) diluted at 1:500 in 5% milk/PBS were then added and incubated for 1 h at 37 °C. After the last three washes, reactions were developed with a 3, 3′, 5, 5′-Tetramethylbenzidine kit (BD OptEIA, BD Biosciences, San Jose, CA, USA), and the optical density was read at 450 nm. Previously well-defined A, B, O, secretor, and Lewis type saliva samples were included in each plate as controls. The cutoff value was defined as a twofold increase in absorbance value compared to the mean of two negative control samples (wells with only PBS). The distribution of ABO blood groups, H type 1 antigen (FUT-2), and Lewis antigens were determined between diarrheic children of the same ages and geographic area infected and non-infected by rotavirus and norovirus.

### 2.5. FUT2 and FUT3 Genotyping

Single nucleotide polymorphisms (SNPs) in the *FUT2* and *FUT3* genes were investigated as described below. Genomic DNA from buccal swabs specimens was extracted with the NucleoSpin^®^Tissue kit (Macherey-Nagel, Düren, Germany), according to the manufacturer’s instructions. For *FUT2* genotyping, a 195 bp *FUT2* gene fragment was initially amplified by PCR and then digested with the enzyme Ava-II, as previously described [39]. This method enables detection of the common G428A *FUT2* mutation as it abrogates the restriction site of the enzyme. For genotyping of the Lewis (*FUT3*) gene mutations, four gene fragments encompassing the worldwide common mutations C314T, G508A, and T1067A were amplified by real-time PCR and submitted to Sanger DNA sequencing, except for mutations T59G and T202C, which were searched for by conventional PCR using sequence-specific primers [40]. For the latter two mutations, a human growth hormone (*hGH*) gene amplification was performed as an internal control in each round of PCR [41]. The *FUT3* sequences involving the C314T/G508A mutations were deposited on GenBank under the accession numbers MT656016-MT656059 and the *FUT3* sequences encompassing T1067A mutation were deposited on GenBank under the accession numbers MT725617-MT725659.

### 2.6. Statistical Analysis

Analysis of categorical data was done with the Fisher exact test, with two-tailed significance using the GraphPad software. A *p* value lower than 0.05 was considered statistically significant.

## 3. Results

### 3.1. Rotavirus and Noroviruses Detection on Children with Acute Diarrhea

Of the 272 children presenting acute diarrhea, 35 (12.9%) and 65 (23.9%) had rotavirus and norovirus infections, respectively (Table 1). Rotavirus and norovirus coinfection was detected in only one child. The age distribution of rotavirus and noroviruses infection can be observed in Table 1.

### 3.2. Rotavirus and Noroviruses Genotyping

The P[8] genotype predominated (30/35; 85.7%) among the rotavirus positive samples, while two (5.7%) belonged to the P[4] genotype and three was P[X]. All the P[8] strains belonged to lineage 3.

Sixty-one out of 65 (93.8%) positive samples for noroviruses belonged to GII, 4.6% to GI, and in one sample (1.5%), both GI and GII were detected. Genotypes were successfully established for 46 samples, and the majority (76%) was represented by the GII.4 Sydney variant (Table 2).

### 3.3. HBGA Phenotypes among Rotavirus and Norovirus Infected Children

Rotavirus predominantly infected children with secretor status (34/35; 97%). The only non-secretor infected child was infected by a P[8] strain. In comparison, 82% (141) of the negative group, which was used as a control group, were secretors, and 18% (31) were non-secretors (*p* = 0.0207) (Table 3). There was no statistical difference between the distribution of Lewis status and ABO blood types among rotavirus infected children compared to the control group (Table 3). Although we observed a lower frequency of Lewis negative children in the infected group as compared with the control group (8.6% vs. 18.5%), the difference was not statistically significant, likely owing to the limited number of cases.

The HBGA secretor phenotype was observed in 64 out of 65 (98.5%) children infected with norovirus, and one infected child (1.5%) had the non-secretor phenotype. Conversely, in the negative control group, 31 out of 172 (18%) children were non-secretors and the difference between the number of non-secretors in cases and controls was statistically significant (*p* = 0.0004) (Table 3). There was no difference in the distribution of ABO blood groups and Lewis status between the norovirus infected individuals and the control group (Table 3). Analysis of HBGA phenotypes stratified by norovirus genotypes showed that all 35 GII.4 strains infected only children with secretor status, and the non-secretor patient was infected by a GII.3 strain (Table 4). The three GI viruses infected the secretor Lewis-positive children, whereas the GII genogroup infected both the secretor Lewis-negative and Lewis-positive children (Table 4).

### 3.4. FUT2 and FUT3 Genotyping

Secretor and non-secretor phenotypes determined by ELISA for both the rotavirus and norovirus infected and the negative control groups were confirmed by *FUT2* genotyping based on the G428A mutation. All non-secretors (*N* = 33) from both the infected and negative groups were homozygous carriers of the *FUT2* G428A nonsense mutation (Table 5). The difference in the number of patients with the non-secretor genotype (se^428^/se^428^) among the rotavirus/norovirus infected groups compared to the negative control group was statistically significant (rotavirus: *p* = 0.0207; norovirus: *p* = 0.0004) (Table 5).

To confirm the Lewis-negative phenotype (Le a and b negative), all 47 samples from children with this phenotype were submitted to *FUT3* gene genotyping, which included the analysis of the common SNPs T59G, T202C, C314T, G508A, and T1067A, and 45 of them were genotypically confirmed as Lewis negative. The most common polymorphism was the T59G with an overall frequency of 86.6% (39/45), followed by the G508A, T202C and C314T SNPs with frequencies of 77.2%, 53.3% and 50%, respectively (Table 6). By contrast, the T1067A SNP was the rarest, with a frequency of 4.6% (2/43), including one homozygote and one heterozygote (Table 6). The frequencies of all SNP analyzed for the *FUT3* gene are shown in Table 7. The two samples with undetermined Lewis genotype had only one heterozygote allele among the investigated mutations. Homozygotes alleles for more than one of the T59G, T202C, C314T, G508A, T1067A mutations were observed in eight individuals, and there were also eight homozygotes for only one mutation. The remaining 29 patients were heterozygote for at least two of the evaluated mutations (Table 7).

## 4. Discussion

The interaction of rotavirus with HBGA has gained importance in the last years, with the discovery of VP8* binding to these glycans [14,15]. Studies have shown that this interaction is P genotype- specific, similar to that observed for VP1 of human noroviruses, and that the susceptibility or resistance to both viruses is largely dependent on the presence or absence of HBGA on gut epithelial surfaces. In addition, the association between the secretor status and infection, initially demonstrated for noroviruses, has also been shown by recent studies for rotavirus, which has demonstrated a correlation between infection and the presence of a functional FUT2 phenotype [23,42,43,44].

In this study, we have shown the association between the HBGA secretor status and rotavirus and noroviruses infection in Brazilian children, a finding that is consistent with previous epidemiological investigations worldwide [18,20,21,22,23,24,25]. Our data add further support to this notion and contribute to the understanding of this association in a highly mixed population like the Brazilian one.

Due to the circulation of emerging P[8]-4 strains [45] with distinct glycan-binding patterns that allow non-secretors to be more prone to infection [46], we searched for possible mutations on the VP8* glycan-binding amino acid sequence of the P[8] strain that infected the non-secretor patient in our study. The analysis of the patient’s VP8* P[8], as well as of the other P[8] strains (all classical P[8]-3 strains) detected in the study, did not demonstrate variations both on conserved and non-conserved residues, compared to other classical P[8] strains detected in the world. Thus, variations on VP8* could not explain the patient’s infection. One hypothesis is that this child was poorly immunized, possibly in part because of his HBGA status (non-secretor), and also because he received only one dose of the Rotarix^®^ vaccine. Later on, the patient may have ingested a large dose of the virus and became ill. We are not dealing with an all or nothing phenomenon and amounts likely matter. This could well be a difference between countries or regions concerning the quality of sanitation and hygiene.

P[8] and P[4] rotavirus genotypes cause more than 90% of the pediatric diarrhea cases in the world, including in Brazil [47]. These data are consistent with those observed in the present study in which all the rotavirus detected belonged to these two genotypes. The fact that P[8] and P[4] genotypes recognize Le^b^ and H type 1 antigens, expressed only in secretor individuals, may explain the worldwide predominance of these genotypes [24,48]. Although the frequency of the secretor phenotype varies across populations, approximately 80% of the world population are secretors [16]. In Brazil, the prevalence of this phenotype revealed in the few studies, is somewhat similar, ranging from 75% to 90% [28,49,50,51]. The 82% of secretors in the control group found in the present study were therefore consistent with these previous observations. The most common *FUT2* mutation, G428A, was observed in all non-secretor children in our study, which contrasts with a recent study performed in North of South America with Amazonian children from Brazil, Venezuela, and English Guyana, where this common *FUT2* gene mutation was not detected [50]. These results highlight the importance of studying the influence of *FUT2* polymorphisms on susceptibility to enteric viruses in populations of different geographic locations, such as those from Latin America.

Apart from studying the susceptibility to natural rotavirus infection, some studies have been focused on the role of HBGA on the rotavirus vaccine response. In this regard, some argue that the differential efficacy of the two main rotavirus vaccines (Rotarix^®^ and RotaTeq^®^) between low-income and high-income countries may be linked to the different HBGA expression in the population. Indeed, higher vaccine take has been observed in children with the secretor phenotype, compared to non-secretors [43,52,53]. Interestingly, in a recent study assessing the association of rotavirus shedding and the HBGA profile in a birth community-cohort in Rio de Janeiro, Brazil, a mutation in the 167 position of the VP8* P[8] gene was detected in many samples characterized as Rotarix^®^ strain (RV1) [54]. Moreover, Le^a+b+^ secretor children were significantly more likely to shed this mutant strain compared to non-secretors. Based on these results, it was suggested that the RV1-vaccine replication triggered by this mutational event led to a more robust immune response in these children [54].

In our study, the majority (72%) of the children were vaccinated against rotavirus, particularly with Rotarix^®^, and this may be considered as introducing a bias, since one may expect non-secretors to be less well vaccinated than secretors. However, rotavirus infected children are still mostly secretors indicating that no such bias occurred, either the secretor status may not have been an important factor in determining vaccine efficacy, or that the most important factor is the genetic susceptibility at the time of infection.

The globally predominant norovirus genotype GII.4, detected in most of our samples (76%), infected only children with the secretor phenotype. These results further confirm the secretor specificity of the GII.4 genotype previously observed through epidemiological studies conducted in different countries such as Burkina Faso, China, Ecuador, USA, Nicaragua, and Vietnam [18,21,55,56,57,58]. Furthermore, among the GII.4, all belonged to the GII.4 Sydney variant which has already been shown to be secretor-specific [18]. Although still unclear, the reasons for the global dominance of GII.4 are at least partially linked to its HBGA-binding characteristics. GII.4 strains bind to glycans of all secretor-positive individuals, irrespective of their ABO and Lewis phenotypes. Moreover, as previously suggested, an increased affinity of HBGA to some of the more recent GII.4 variants may have contributed to the epidemiological dominance of these variants [59]. It is conceivable that these features, in addition to the high prevalence of secretor-positive individuals in the world population, would provide a broad spectrum of susceptible hosts worldwide and affect GII.4 infection.

Regarding the non-GII.4 norovirus genotypes, all but one was found among the infected secretor-positive children, although we detected GII.1 and GII.2 genotypes capable of infecting non-secretors to a similar extent as secretors [57,60]. The only non-secretor child was infected with the GII.3 norovirus genotype. In fact, this genotype has been reported to infect both secretors and non-secretors, and although some studies indicate that the secretors are more prone to infection by that genotype, the association is not as strong as that observed for the GII.4 genotype [21,61].

We observed a trend towards the occurrence of rotavirus infection on secretor Lewis positive children (91.4%), without reaching statistical significance, probably due to the low number of rotavirus cases. This result is similar to that obtained in an epidemiological study conducted in Burkina Faso and Nicaragua that showed that no secretor/Lewis negative child with diarrhea was infected by P[8] [22]. Moreover, previous studies suggest that both the Lewis b and secretor antigens are required for P[8] and P[4] VP8* binding [24,48,62]. On the other hand, recent structural studies involving P[8] VP8* binding to HBGA presented conflicting results. While some indicate the additional fucose conferred by the Lewis b antigen would cause steric hindrance to the interaction, other studies demonstrated binding of P[8] VP8* to the Lewis b antigen via a pocket formed by two β-sheets [63,64,65]. Further studies are warranted to clarify this issue.

Overall, the results of the Lewis phenotyping revealed that the Lewis status (positive/negative) is not a mediator of susceptibility to norovirus infection, an observation consistent with previous studies [55,58,66]. Nevertheless, one study from Taiwan observed that the Lewis antigen-positive genotype was a protective factor against severe norovirus gastroenteritis [67]. In addition, while the three norovirus GI infected only Lewis-positive children, the norovirus GII infected both Lewis-negative and Lewis-positive children, similar to previous findings from Burkina Faso [55]. In favor of the GI’s preference to infect Lewis-positive individuals, Kubota and colleagues demonstrated a structural basis for the recognition of Lewis antigens by the GI norovirus [68]. Unfortunately, due to the limited number of GI positive samples, we could not evaluate the Lewis effect for individual genotypes.

The frequency of 17.1% of Lewis-negative individuals observed in the present study is higher than that registered in people of European descent such as in Scandinavia (5.7%), Spain (6%), and Portugal (10%) [25,66,69]. Nevertheless, the frequency of this phenotype in the highly mixed Brazilian population is similar to other countries of the American continent including Colombia (22%) and Nicaragua (25%), and can reach up to 32% in some African populations [22,58,70]. Among the Lewis negative individuals, four main SNP were identified at positions T59G, G508A, T202C, and C314T. These results are in agreement with a study conducted with an Amazonian population, and a recent birth community-cohort in Rio de Janeiro, in which these SNPs were detected with similar frequencies [29,71]. In addition, the combination of SNPs found in the present study are also known to occur in Asian, African, and European populations, with the T59G and G508A being more frequent in the first two continents, and the T202C and C314T in the latter [72,73,74].

## 5. Conclusions

Our findings add to the existing knowledge that children with an *FUT2* secretor status are indeed more susceptible to both rotavirus and norovirus symptomatic infection, compared to non-secretors, and contribute to improving the understanding of the host susceptibility to these viruses and their epidemiology in Latin America. Moreover, the high rate for Lewis negative and the combination of SNPs, beyond the secretor status, may reflect the highly mixed population from Brazil, and, to some extent, restrict the infection by rotavirus but not by norovirus. New studies in populations with distinct ethnicities from Brazil and other countries of the South American continent are still warranted to better evaluate the impact of *FUT2* and *FUT3* polymorphisms on rotavirus and norovirus susceptibility.

## Figures and Tables

**Table 1:** Distribution of children infected with rotavirus and noroviruses according to age groups.

Age	Number of Samples *N* (%)	Rotavirus+ *N* (%)	Norovirus+ *N* (%)	Negative *N* (%)
≤12 m	99 (36.4)	9 (9.1)	33 (33.3)	57 (57.6)
>12 m–2 y	78 (28.7)	9 (11)	20 (25)	50 (64)
>2–5 y	61 (22.4)	13 (21.3)	9 (14.7)	39 (64)
>5 y	34 (12.5)	5 (14.7)	3 (8.8)	26 (76.5)
Total	272	35 (12.9)	65 (23.9)	172 (63.2)

Abbreviations: m, months; y, years; Rotavirus+, rotavirus infected children; Norovirus+, norovirus infected children.

**Table 2:** Noroviruses *VP1* gene genotyping.

VP1	Strains *N* (%)
GI.7	1 (2.2)
GII.4 Sydney	35 (76)
GII.17	5 (10.9)
GII.3	2 (4.3)
GII.1	1 (2.2)
GII.2	1 (2.2)
GII.6	1 (2.2)

**Table 3:** Distribution of histo-blood group antigens (HBGAs) phenotypes between children infected with rotavirus/noroviruses and the negative control group.

HBGA Phenotype	Total *N* (%)	Rotavirus+ *N* (%)	Norovirus+ *N* (%)	Negative Control Group *N* (%)	*p* Value	
					Rotavirus+	Norovirus+
**Secretor status:**					0.0207	0.0004
Se	239 (87.9)	34 (97)	64 (98.5)	141 (82)		
se	33 (12.1)	1 (3)	1 (1.5)	31 (18)		
**Lewis phenotype:**					0.2154	1.0000
Le^+^	225 (82.5)	32 (91.4)	53 (81.5)	140 (81.4)		
Le^−^	47 (17.1)	3 (8.6)	12 (18.5)	32 (18.6)		
**Blood groups:**					0.4464	0.2907
O	128 (53.6)	15 (44)	39 (61)	74 (52.5)		
A	72 (30.1)	11 (32.4)	19 (29.7)	42 (29.8)		
B	31 (13)	6 (17.6)	4 (6.2)	21 (14.9)		
AB	8 (3.3)	2 (6)	2 (3.1)	4 (2.8)		

Abbreviations: Le^+^, Lewis-positive (Lewis a and/or b positive); Le^−^, Lewis-negative (Lewis a and b negative); Norovirus+, norovirus infected children; Rotavirus+, rotavirus infected children; Se, secretor; se, non-secretor.

**Table 4:** Distribution of histo-blood group antigen phenotypes in relation to noroviruses genogroup and genotypes among children infected with noroviruses in Espírito Santo state, Brazil.

Norovirus Genogroup/Genotype	*N*	Blood Groups				Lewis Phenotypes			Secretor Status	
		O	A	B	AB	Le^a−b+^	Le^a−b−^	Le^a+b−^	Secretor	Non-Secretor
G.I *	3	1	1	1	-	3	-	-	3	-
G.II	61	39	15	4	2	50	10	1	60	1
GII.1	1	1	-	-	-	1	-	-	1	-
GII.2	1	1	-	-	-	1	-	-	1	-
GII.3	2	-	1	-	-	1	-	1	1	1
GII.4	35	24	9	1	1	27	8	-	35	-
GII.6	1	-	1	-	-	1	-	-	1	-
GII.17	5	2	3	-	-	3	2	-	5	-
GII *	16	11	1	3	1	16	-	-	16	-
G.I and G.II	1	-	1	-	-	-	1	-	1	-

Abbreviations: Le^a−b+^, Lewis a negative and Lewis b positive; Le^a−b−^, Lewis-negative (Lewis a and b negative); Le^a+b−^, Lewis a positive and Lewis b negative. * Refers to strains with undetermined genotypes.

**Table 5:** Distribution of *FUT-2* genotypes among infected patients and controls according to G428A mutation.

*FUT-2* Genotype	Total *N* (%)	Rotavirus+ *N* (%)	Norovirus+ *N* (%)	Negative Control Group *N* (%)	*p* Value	
					Rotavirus+	Norovirus+
SE/SE	99 (36.4)	11 (31.4)	33 (50.8)	55 (32)	0.0207	0.0004
SE/se^428^	140 (51.5)	23 (65.7)	31 (47.7)	86 (50)		
se^428^/se^428^	33 (12.1)	1 (2.9)	1 (1.5)	31 (18)		

Abbreviations: *FUT-2*, fucosyltransferase 2 gene; SE/SE, homozygote secretor; SE/se^428^, heterozygote secretor; se^428^/se^428^, non-secretor genotype; Norovirus+, norovirus infected children; Rotavirus+, rotavirus infected children.

**Table 6:** Frequencies of polymorphisms on *FUT-3* gene in Lewis negative children.

SNP	Frequency, *N* (%)			
	Mutant Homozygotes	Heterozygotes	Wild Type Homozygotes	Total
G508A	10 (22.7)	24 (54.6)	10 (22.7)	44 ^a^
T59G	8 (17.8)	31 (68.9)	6 (13.3)	45
T202C	3 (6.6)	21 (46.7)	21 (46.7)	45
C314T	2 (4.5)	20 (45.5)	22 (50)	44 ^a^
T1067A	1 (2.3)	1 (2.3)	41 (95.4)	43 ^a^

Abbreviations: SNP, single nucleotide polymorphism. ^a^ One sequence for one patient for mutations G508A and C314T, and two sequences for T1067A could not be determined.

**Table 7:** *FUT3* alleles frequencies observed in South-eastern Brazilian children.

*FUT3* Allele	Frequency, *N* (%)
*Lele^59,202,314,508^*	13 (31)
*Lele^59,508^*	11 (26)
*Lele^202,314^*	4 (9.5)
*Lele^59,202,314,1067^*	1 (2.4)
*Lele^59,202,314^*	1 (2.4)
*Lele^59,202^*	1 (2.4)
*le^508^le^59,508^*	5 (11.9)
*le^59^le^59,508^*	2 (4.8)
*le^59,1067^le^59,202,1067^*	1 (2.4)
*le^202^le^59,202,314^*	1 (2.4)
*le^59,508^le^59,508^*	1 (2.4)
*le^202,314^le^202,314^*	1 (2.4)

Abbreviations: *Le*, Lewis wild-type allele; *le*, Lewis mutant allele.
